# On the Properties of Nafion Membranes Recast from Dispersion in *N*-Methyl-2-Pyrrolidone

**DOI:** 10.3390/polym14235275

**Published:** 2022-12-02

**Authors:** Ekaterina Yu. Safronova, Daria Yu. Voropaeva, Anna A. Lysova, Oleg V. Korchagin, Vera A. Bogdanovskaya, Andrey B. Yaroslavtsev

**Affiliations:** 1 N. S. Kurnakov Institute of General and Inorganic Chemistry, Russian Academy of Sciences, 119991 Moscow, Russia; 2 A. N. Frumkin Institute of Physical Chemistry and Electrochemistry, Russian Academy of Sciences, 119991 Moscow, Russia

**Keywords:** Nafion membrane, membrane casting, proton conductivity, fuel cell

## Abstract

Perfluorosulfonic acid Nafion membranes are widely used as an electrolyte in electrolysis processes and in fuel cells. Changing the preparation and pretreatment conditions of Nafion membranes allows for the optimization of their properties. In this work, a Nafion-NMP membrane with a higher conductivity than the commercial Nafion^®^ 212 membrane (11.5 and 8.7 mS∙cm^−1^ in contact with water at t = 30 °C) and a comparable hydrogen permeability was obtained by casting from a Nafion dispersion in *N*-methyl-2-pyrrolidone. Since the ion-exchange capacity and the water uptake of these membranes are similar, it can be assumed that the increase in conductivity is the result of optimizing the Nafion-NMP microstructure by improving the connectivity of the pores and channels system. This leads to a 27% increase in the capacity of the membrane electrode assembly with the Nafion-NMP membrane compared to the Nafion^®^ 212 membrane. Thus, the method of obtaining a Nafion membrane has a great influence on its properties and performance of fuel cells based on them.

## 1. Introduction

The 21st century challenges of the negative environmental and climate impacts of fossil-fuel energy generation, as well as the rapid depletion of fossil-fuel resources, requires an increase in renewable and alternative energy sources. [1,2]. A switch to green energy derived from natural sources (biomass, solar, wind, water, etc.) is limited due to the irregularity of its production over time [3]. In this regard, research and development aimed at creating new types of energy storage becomes more attractive. Proton-exchange membrane fuel cells (PEMFCs) have a number of advantages in comparison with the conventional technologies based on fuel combustion, as well as with other energy storage systems. PEMFCs have a high efficiency, the lowest emissions, and are practically free from vibration and noise during their operation [4,5].

One of the main components determining the efficiency of PEMFCs is the membrane separating the cathode and anode sides, which ensures a rapid proton transfer and prevents the migration of non-polar molecules and electrons [6,7]. The most appropriate materials in such PEMFCs are Nafion-type membranes, representing by polytetrafluoroethylene with relatively long side chains formed by the ether and fluorocarbon groups terminated with functional sulfonic groups (-O-CF_2_-CF(CF_3_)-(CF_2_)_2_-SO_3_H) [8]. The commercially available Nafion^®^ 212 membrane is the material most commonly used in PEMFCs. Its high conductivity and selectivity of cation transport, low gas permeability, and electronic conductivity along with its good mechanical properties and chemical stability has led to the interest of researchers in this material. However, along with these advantages, Nafion^®^ 212 membranes have drawbacks, including a sharp decrease in ionic conductivity at a low humidity, a limited operating temperature range (t < 100–120 °C) due to a reduced conductivity, a loss of mechanical strength at high temperatures, and a high cost.

The properties of perfluorosulfonic acid membranes are determined by their microstructure [9]. The understanding of the microstructure is based on the comparison of the results of small-angle X-ray and neutron scattering with the transport and sorption properties of the membranes [9,10]. The differences in the nature of the main and side chains of the polymer lead to the clustering of hydrophilic -SO_3_H groups. Their hydration ensures the formation of pores and channels system in the membrane, through which the ionic transport is carried out [11,12]. The microstructure of perfluorosulfonic acid membranes, their ionic conductivity, and the selectivity of their transport processes are largely determined by their water uptake [13,14]. In addition, the membrane microstructure is sensitive to many environmental conditions including changes in the temperature, humidity, mechanical deformation, composition, and concentration of solutions in contact with it [15,16,17,18,19]. Nafion-type membranes have a memory effect, and their properties can be changed by a preliminary treatment. The irreversibility of the processes that occur during treatment is related to the conformational transformations of the polymer when heated and their inhibition at room temperature.

The use of thin films (less than 50 μm) as an electrolyte in the PEMFCs reduces the resistivity and increases the power of the membrane electrode assembly (MEA) [5]. The optimal way to create such a thick membrane is to cast it from a polymer solution or dispersion. The film’s thickness affects the specific conductivity and water uptake of the membranes [20]. When Nafion membranes are casting, their microstructure depends strongly on the conditions under which the films are produced (dispersing liquid, speed, and temperature of the solvent removal) [21,22,23,24]. The morphology of the polymer in the dispersion depends on the nature of the dispersing liquid. It was shown that in the process of solvent removal from Nafion dispersions, gelation occurs at different polymer concentrations, and its mechanism as well as the mechanical properties of the casting membranes also differs depending on the solvent nature [25]. The most durable are membranes cast from dispersions in aprotic solvents, such as *N,N*-dimethylformamide and *N*-methyl-2-pyrrolidone. In contact with aprotic solvents, polymers do not agglomerate and are most similar to the solution state [22]. This ensures the formation of a well-defined bond network and, as a result, films with good mechanical properties [25]. The ionic conductivity of perfluorosulfonic acid casting membranes also varies depending on the nature of the solvent [26,27]. However, most of the works investigating the properties of Nafion membranes obtained from various dispersions are devoted to their application as a binder in the catalytic layer [18,28,29,30,31,32], and the development of membranes with improved properties for their use as an electrolyte seems promising.

The aim of this work was the investigation of the transport and mechanical properties of the Nafion-NMP membrane obtained from the polymer dispersion in the aprotic solvent *N*-methyl-2-pyrrolidone and characteristics of PEMFC based on it and their comparison with the commercial Nafion^®^ 212 membrane. The choice of aprotic solvent as a dispersing liquid was due to the possibility of obtaining films with good mechanical properties and a uniform morphology. The low vapor pressure of *N*-methyl-2-pyrrolidone allows for obtaining the film at a low temperature.

## 2. Materials and Methods

The following materials and reagents were used: Nafion^®^ 212 membrane (equivalent weight 1100, the Chemours Company, Wilmington, DE, USA), 5% solution of Nafion^®^ in low-molecular weight alcohols (equivalent weight 1100, The Chemours Company, Wilmington, DE, USA), Sigraget 39 BB (SGL Carbon, Bonn, Germany), *N*-methyl-2-pyrrolidone (Merck, Darmstadt, Germany), lithium hydroxide (LiOH, ≥99.0%, Sigma Aldrich, St. Louis, MO, USA), hydrochloric acid (special purity, 35–38%, Chimmed, Moscow, Russia), potassium chloride (reagent grade, Chimmed, Moscow, Russia), potassium hydroxide (reagent grade, Chimmed, Moscow, Russia), and deionized water (resistance 18.2 MOhm).

To obtain the polymer dispersion in *N*-methyl-2-pyrrolidone, a Nafion^®^ 212 membrane in Li^+^ form was used. A commercial Nafion^®^ 212 membrane was preliminary conditioned under standard conditions: treated at 80 °C in 3 wt.% H_2_O_2_, 80 °C in 5 wt.% HCl solution for 1.5 h, and washed with deionized water at 90 °C until the reaction for the Cl^−^ ions was ceased [33]. The conversion to Li^+^ form was performed by soaking the membrane in 1M LiOH solution with a threefold replacement of the solution. The use of the polymer in Li^+^-form is due to increases in the thermal stability of the polymer with alkali counterions in comparison to the protons. To remove hydroxide ions from the membrane, it was washed with deionized water until reaching a neutral pH. The obtained sample was dried in an OV-11/12 vacuum oven (Jeio tech, Daejion, Republic of Korea) at 50 °C for 6 h. The film was crushed and placed in a solvothermal cell. *N*-methyl-2-pyrrolidone was added in a value to obtain a dispersion with 5 wt.% of the polymer. The mixture was kept at 100 °C for 2 h.

For the formation of the Nafion-NMP membrane, the obtained dispersion was cast on a glass surface (Petri dishes with the diameter of 11 cm) and heated to remove the solvent (60 °C for 3 h in air, 120 °C for 6 h under vacuum). The obtained films were removed from the glass surface and subjected to hot pressing at 5 MPa at 110 °C for 3 min to ensure a better strength. The films were conditioned to the standard conditions and transferred into protonic form. For this purpose, they were successively treated at room temperature twice with a 5 wt.%. HCl solution for 1.5 h, then was washed with deionized water until the reaction with Cl^−^ ions disappeared. The membrane obtained from the dispersion in *N*-methyl-2-pyrrolidone is further designated as Nafion-NMP. The comparison sample was the native Nafion^®^ 212 membrane. Both membranes were 52 ± 3 μm thick in the dry state. The samples were stored in deionized water. The properties of the membranes in H^+^−form were studied.

The dispersion density was determined using a Densito portable density meter (Mettler Toledo, Greifensee, Switzerland) at 25 ± 0.1 °C. The viscosity of the dispersion was determined using a vibro viscometer SV-1A (A&D, Tokyo, Japan) at 25 ± 0.2 °C. The value of the dynamic viscosity (η, mPa∙s) was calculated from the ratio of the experimentally obtained viscosity to the density of the dispersion. The viscometer was calibrated at two points using 5 and 10 mPa∙s viscosity standards (Brookfield, Toronto, ON, Canada). The viscosity was calculated from the average of the three independent experiments.

The IR spectra of the membranes in a dry state were recorded on a Nicolet iS5 FTIR spectrometer (Thermo Scientific, Waltham, MA, USA) with the Fourier transformation and ATR add-on (a diamond crystal).

Differential scanning calorimetry (DSC) was performed on a STA 449F1 (Netzsch, Wunsiedel, Germany) in aluminum crucibles under a helium atmosphere at a flow rate of 20 mL∙min^−1^. The samples were previously dried in a vacuum oven at 50 °C for 6 h for dehydration. The analysis was performed in three steps: (i) preheating from 25 to 80 °C with holding at 80 °C for 30 min to remove the water sorbed from the surface; (ii) cooling from 80 to −130 °C; and (iii) heating from −130 to 240 °C. The heating/cooling rate was 10 °C∙min^−1^. The maximum DSC analysis temperature was due to the onset of the thermal decomposition of the polymer, which can lead to the contamination and failure of the apparatus.

The mechanical properties of the membranes were examined using an H5KT tensile test machine (Tinius Olsen, Horsham, PA, USA) with a 100 N force transducer at 25 ± 2 °C and a relative humidity RH = 20 ± 2%. The tensile rate was 5 mm/min. The films were held at a relative humidity of RH = 32% before being placed into a desiccator under a saturated CaCl_2_ solution. Rectangular samples with a length of 70 mm (gauge length was 45 mm) and a width of 7 mm were used. Five experiments were performed for each membrane. The thickness and width were determined immediately before the experiment as an average of 5 points along the entire length (using a Mitutoyo micrometer, 0.001 mm accuracy). Young’s modulus was determined from the slope of the stress–strain curve in the elastic strain region. The mean values were calculated for each series of samples, and the error of measurement was evaluated by Student’s t-distribution.

The ion-exchange capacity (IEC, mg-eq∙g^−1^) of Nafion^®^ 212 and Nafion-NMP membranes was determined by direct acid-base titration using an Econix Expert 001 pH meter (Econix-Expert, Moscow, Russia) to establish the equivalence point. The H^+^-form membranes were previously kept at 150 °C for 30 min to remove the water. A sample weight of ~0.3 g was placed in 50 mL of 0.1M NaCl solution for 6 h under constant stirring. After that, the salt solution with the membrane was titrated with 0.05 M of NaOH solution. The IEC was calculated relative to the weight of the dry membrane in H^+^-form.

For the water uptake, the proton conductivity and gas permeability of at least 3 samples of one type of membrane were tested. The mean values were calculated for each series, and the error of measurement was evaluated by Student’s t-distribution.

The water uptake of the membranes was determined using TG 209 F1 (Netzsch, Wunsiedel, Germany) after their long-term contact with water and after a previous equilibration at a humidity of RH = 32%. The amount of water in the sample was calculated as the difference in the mass before the heat treatment and after keeping at 150 °C to the mass of the sample before the heat treatment.

The proton conductivity of the membranes was studied at the humidity of RH = 100% in contact with deionized water in the temperature range 25–85 °C and in air at a humidity of RH = 30% in the temperature range 25–60 °C. A climate chamber MKF115 (Binder, Tuttlingen, Germany) was used to set the necessary humidity (humidity setting accuracy ± 2.5%) and temperature. The measurements were performed using an AC bridge E-1500 (Elins, Chernogolovka, Russia) in the frequency range of 10 Hz to 3 MHz on symmetrical carbon/membrane/carbon cells with an active surface area of 1 cm^2^. The values of conductivity (Ohm^−1^∙cm^−1^) were calculated from the resistance obtained from the impedance hodographs at the intercept with the active resistance’s axis. The accuracy of the conductivity was less than 10%.

The hydrogen permeability of the membranes was determined by gas chromatography using a Crystallux-4000M chromatograph (NPF Meta-chrom, LTD, Mari El Republic, Yoshkar-Ola, Russia) with a thermal conductivity detector (30 mA current) and a packed column (Mole Seive 5 Å, 2 m, 30 °C, 20 cm^3^∙min^−1^, Ar) at RH = 95% humidity. To produce hydrogen, a pure hydrogen source GVCh-12A (Khimelektronika Ltd., Moscow, Russia) was used. The experiment was carried out in a thermo-stated two-chamber cell with an active surface area of 4 cm^2^. Pure hydrogen was fed into one chamber and argon was fed into the other chamber at the rate of 20 mL∙min^−1^. To create the necessary level of humidity in the flow of hydrogen and argon, each gas was passed through two water tanks, which were thermostatically controlled at the same temperature as the cell. The hydrogen permeability coefficient *P* (cm^2^∙s^−1^) was calculated according to the following equation:

(1) $$P=\frac{jL}{C_{H}-C_{Ar}},$$

where L is the membrane thickness, $C_{H}$ is the average volume concentration of the hydrogen in the chamber in which hydrogen was supplied, and $C_{Ar}$ is the average volume concentration of hydrogen in the chamber in which argon was supplied. The gas flow through the membrane (j) was calculated from the equation:

(2) $$j=\frac{C_{Ar}V_{t}}{S},$$

where $V_{t}$ is the volumetric velocity of the flow of the carrier gas and S is the active surface area of the membrane.

The MEAs was formed as follows. Catalytic inks were applied on the surface of the membrane from both sides by using an airbrush. The surface area of the catalytic layer was 1 cm^2^. The catalytic inks were prepared from a water–alcohol dispersion of the catalyst and Nafion binder (5 wt.% solution of Nafion^®^ in low-molecular-weight alcohols). A 40 wt.% Pt/carbon nanotube (CNT) catalyst was used, which was applied in an amount of 0.6 mg_Pt_∙cm^−2^ to the cathode and 0.3 mg_Pt_∙cm^−2^ to the anode. The synthesis technique and characteristics of this catalyst are presented in the work [34]. The mass ratio of the Nafion binder (by the dry matter) to the mass of the catalyst support (CNT) was 0.9. The membrane with the applied catalytic layers was sandwiched in an ElectroChem test cell (Electrochem Headquarters, Raynham, MA, USA) between two Sigraget 39 BB gas diffusion layers. The compression degree of the MEA was 20–25% and was controlled by adjusting the thickness of the Teflon spacers.

The MEAs were tested using a specialized ElectroChem fuel test station (Electrochem Headquarters, Raynham, MA, USA). Electrochemical measurements were performed using an Elins P-45x potentiostat-galvanostat with an FRA module (Elins, Chernogolovka, Russia). The voltametric characteristics were determined under near-stationary conditions by supplying the cell with hydrogen (in the anode space) and oxygen (in the cathode space) without overpressure, with the humidification corresponding to the relative humidity RH~100%. The cell temperature was 65 °C. The electrochemically active surface area of the platinum catalyst (S_Pt_) in the cathode was determined from the cyclic voltammograms recorded at 50 mV∙s^−1^ under nitrogen purging conditions of the cathode space. The catalyst areas in the MEAs based on Nafion^®^ 212 and Nafion-NMP had close values and were S_Pt_ = 25 and 22 m^2^∙g_Pt_^−1^, respectively. The difference in the voltage determination for 2 MEAs based on one type of membrane was less than 5%.

## 3. Results and Discussions

It is known that the morphology and the properties of perfluorosulfonic acid membranes largely depend on the method of their formation. In particular, in the case of casting films, the nature of the dispersing liquid affects the arrangement of the membrane’s inner space and its properties [25]. When aprotic solvents, such as *N*-methyl-2-pyrrolidone, are used, the morphology of Nafion in the dispersion is most similar to the solution, the macromolecules are not aggregated, and they represent random coils several nanometers in size. In this case, the side chains have a high mobility [22]. The viscosity of the Nafion dispersion in *N*-methyl-2-pyrolidone was 11.5 mPa∙s, while the viscosity of the native solvent itself was 4.1 times lower, which indicates an effective polymer–solvent interaction.

**Figure 1:**
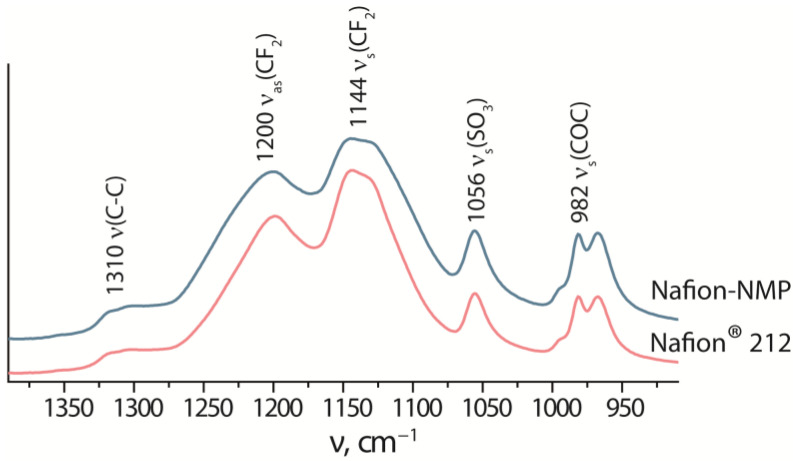
IR spectra of the dry Nafion^®^ 212 and Nafion-NMP membranes.

According to the IR spectroscopy data, there are no changes in the position and intensity of the vibrational bands of the Nafion^®^ 212 and Nafion-NMP membranes (Figure 1). The IEC of Nafion^®^ 212 (0.95 mg-eq∙g^−1^) and Nafion-NMP (0.94 mg-eq∙g^−1^) membranes has similar values. Thus, it can be assumed that there is no degradation of the polymer under the conditions of obtaining the dispersion in *N*-methyl-2-pyrolidone.

**Figure 2:**
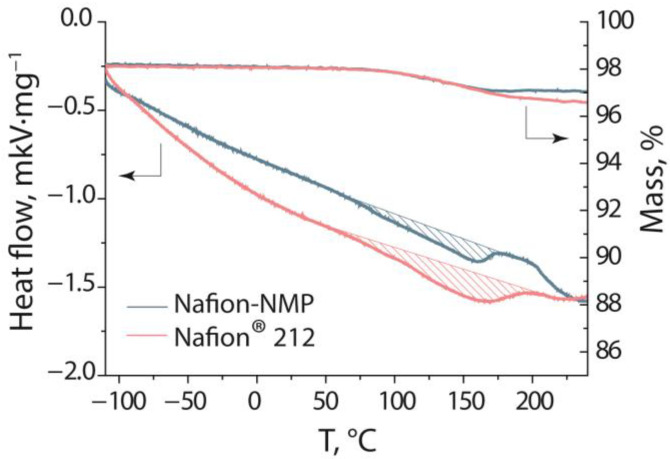
DSC curves and mass loss as a function of temperature for Nafion^®^ 212 and Nafion-NMP dry membranes (indicated in the figure).

The DSC curves of Nafion^®^ 212 and Nafion-NMP membranes show one endothermic peak and the beginning of the second endothermic peak (Figure 2), which are typical for Nafion-type membranes [35,36]. The beginning of the first peak is at 90 °C and can be attributed to the dehydration of the membranes. The Nafion membranes retain 1–2 water molecules up to 200 °C, which are removed only with the decomposition of the sulfonic groups [37]. Even though the samples were previously dried under a vacuum at 50 °C, at the first stage of the DSC experiment at 80 °C, their mass decreased by 1.8 wt.% due to the evaporation of water absorbed on the surface. The mass loss of Nafion^®^ 212 and Nafion-NMP membranes in the third step of the DSC experiment during heating from −130 to 240 °C differs and is 1.4 and 0.9 wt.%, respectively. Some of the differences in the observed thermal effects are related to changes in the microstructure of the membranes. The second thermal effect is attributed to the processes occurring in the hydrophobic matrix and is associated with the melting of the crystalline parts of the ionomer [35]. In the case of both investigated membranes, the temperature at the beginning of the second heat effect is ~200 °C. Thus, it can be concluded that the casting of the Nafion-NMP membrane under laboratory conditions does not lead to a significant change in its thermal stability compared to Nafion^®^ 212.

**Figure 3:**
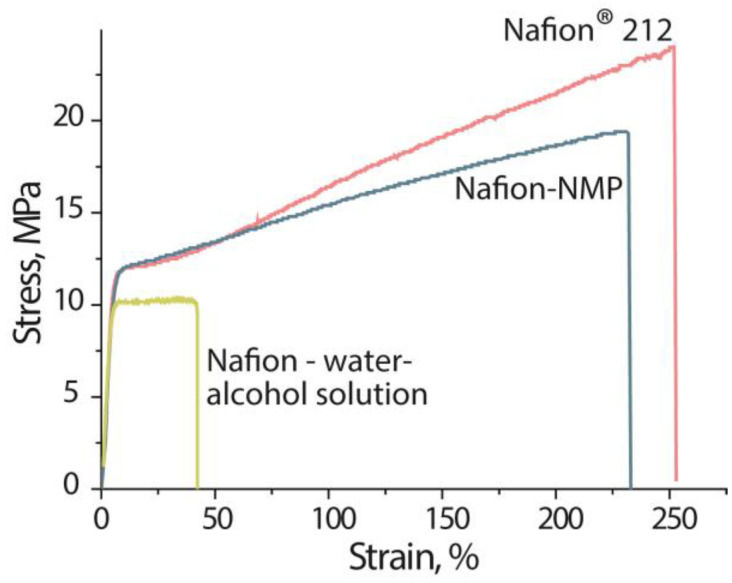
Stress–strain curves of Nafion^®^ 212, Nafion-NMP membranes kept at RH = 32% in comparison with membrane obtained via casting procedure from water–alcohol solution of Nafion, described in [38].

The stress–strain curves for Nafion^®^ 212 and Nafion-NMP membranes are comparable (Figure 3). The mechanical properties of the membranes are shown in Table 1. The break strain and the proportional limit stress of Nafion^®^ 212 and Nafion-NMP membranes are the same within the margin of error. Casting the membranes from the dispersion in *N*-methyl-2-pyrrolidone results in high-strength materials, although with slightly lower values of break stress and Young’s modulus compared to the Nafion^®^ 212 membrane. The lower strength of the Nafion-NMP membrane may be related to the features of the solvent’s removal under laboratory conditions compared to industrial ones, as well as with the composition of the dispersion. In general, the strength of the membrane obtained from the dispersion in *N*-methyl-2-pyrrolidone is significantly higher than the membrane obtained by casting from the water–alcohol solution of Nafion, for which the break stress and break strain are 2 and 5.8 times lower [38]. The peculiarities of perfluorosulfonic acid polymer’s morphology in contact with an aprotic solvent ensures the formation of a well-connected polymer matrix and leads to the formation of membranes with a high durability. The reason for this is that in a solution of perfluorosulfonic acid membranes in an aprotic solvent, the external shell of the polymer globules is formed by hydrophobic polymer chains, which are easily bonded when drying. On the contrary, in a water–alcohol solution of Nafion, hydrophilic functional groups are released on the surface of the globules and the formation of the membrane is complicated. According to [22], the polymer in this case tends to the agglomeration and the mobility of the side chains is limited compared to the dispersions in aprotic solvents. The Young’s modulus of the membranes obtained from the water–alcohol solutions and dispersions in *N*-methyl-2-pyrrolidone are the same (Table 1). Young’s modulus corresponds to the stiffness of the membranes and depends primarily on the strength of the electrostatic interactions within the hydrophilic pores [39,40]. Since the number of functional groups in Nafion-NMP and Nafion membranes obtained from a water–alcohol solution of the polymer (Nafion–water–alcohol solution) are the same, Young’s modulus has similar values.

Among the performance requirements for membranes for PEMFCs, a high proton conductivity and low gas permeability are the most important for achieving high power densities [41]. The gas permeability determines the hydrogen crossover from the anodic to the cathodic space, which is not accompanied by the generation of electricity and leads to a decrease in the operating potential of PEMFCs. The transport properties of perfluorosulfonic acid membranes are determined by the pores and channels system. The pores are formed due to the self-organization of the polymer during the membrane formation, owing to differences in the nature of the main chain and the side groups of the membrane [9]. The high affinity of the sulfonic groups for hydration leads to water sorption, an increase in the pore size, and the appearance of interconnecting channels [12]. A proton transfer occurs mainly close to the pore walls within the narrow Debye’s layer formed by the functional groups [42,43]. In this case, the mechanism of their transfer, the values of conductivity, and its activation energy depend on the water content and the size of the pores and channels [42,44]. The transfer of non-polar molecules, such as hydrogen, is predominantly carried out through the electroneutral solution in the center of the pores and partially through the polymer matrix [13,45]. Important factors determining transport in perfluorosulfonic acid membranes are their water uptake and morphology (size and interconnectivity) of the pores and channels [42].

A direct visualization of the microstructure of the Nafion membranes using electron microscopy is impossible for several reasons. In electron microscopy, the resolution of the image is determined not by instrumental parameters, but by the sensitivity of the material to radiation. Under the action of a high-energy electron beam, membranes are partially or completely destroyed; therefore, images of such materials are obtained with a low signal-to-noise ratio [46]. Another important problem is that high-resolution studies are carried out in a vacuum, and under these conditions, hydrophilic pores disappear. Studying the morphology of Nafion membranes using scanning electron microscopy is also difficult due to the low resolution and weak contrast between the hydrophobic matrix and ion clusters [46]. The microstructure of Nafion membranes was proposed on the basis of small-angle neutron scattering and small-angle X-ray scattering data and their correlation with the water uptake and ion transport [9,12,47]. Based on this, the difference in the morphology of the membranes can be assessed indirectly, based on changes in such characteristics as the mechanical properties, water uptake, ionic conductivity, etc.

**Figure 4:**
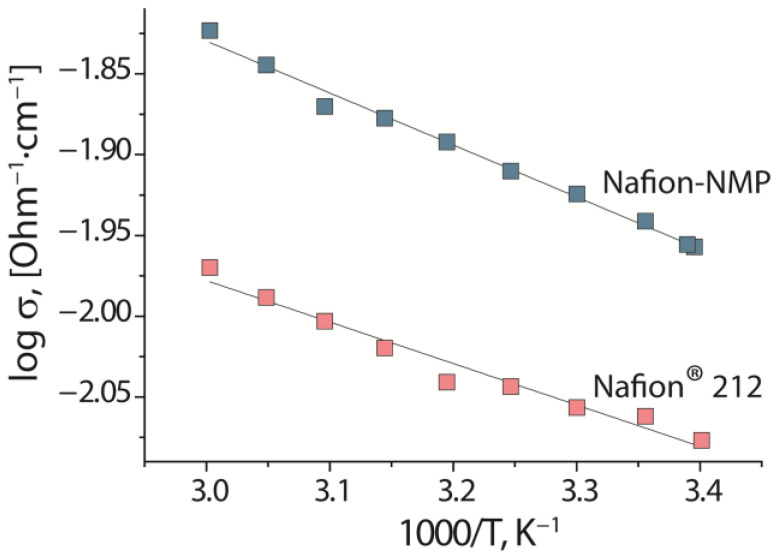
Temperature dependence of proton conductivity of Nafion^®^ 212 and Nafion-NMP membranes in contact with water.

The water uptake of the membranes in contact with water is determined by the number of functional groups and the possibility of a pore enlargement during hydration, so it practically does not differ for the investigated membranes (Table 2). When producing membranes from dispersion in *N*-methyl-2-pyrolidone, the optimal solvent removal temperature was found to be 120 °C, although the boiling point of the solvent is much higher (202 °C). This accounts for its low evaporation rate, which also contributes to the reorientation of the polymer chains and their arrangement in the most favorable way. This, along with the favorable orientation of the globules in the dispersion and a lower degree of agglomeration in contact with *N*-methyl-2-pyrrolidone promotes the formation of films with a uniform pore size and a good interconnectivity. Such morphological changes in the polymer during casting results in the rapid proton transport and a higher proton conductivity of the Nafion-NMP membrane compared to Nafion^®^ 212 in contact with water (Table 2, Figure 4). The increase in the conductivity of the Nafion-NMP membrane is 30%, as compared to Nafion^®^ 212. The conductivity is determined by the product of the number of current carriers on their mobility. As the number of current carrier and water uptake are equal for the Nafion-NMP and Nafion^®^ 212 membranes, the rise in the conductivity is caused by the high proton mobility. Proton transport in perfluorosulfonic acid membranes at high hydration level occurs by the Grotthuss mechanism and is determined by the transfer through the narrow channels [42,48]. The improvement in the microstructure and connectivity of the pores results in the increase in the proton conductivity of the Nafion-NMP membrane.

The great advantage of the Nafion-NMP membrane is the higher water uptake at a low humidity (RH = 32%, Table 2) because it protects the electrolyte from a sharp conductivity drop when water is transferred from the anode space with protons during the PEMFC operation.

**Figure 5:**
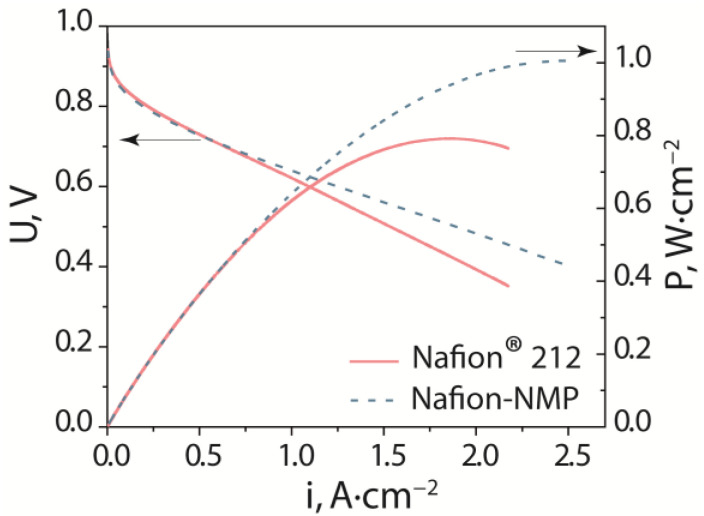
Voltammetry curves and power density dependence on current density for MEAs (RH = 100%, t = 65 °C).

The hydrogen gas permeability of the membrane is also an important characteristic for PEMFCs. The ratio of the gas permeability to proton conductivity determines the selectivity of the membranes in PEMFCs. The gas permeability of Nafion^®^ 212 and Nafion-NMP membranes varies within the margin of error (Table 2). Thus, the higher the conductivity of the Nafion-NMP membrane, the higher the performance in the PEMFCs. Figure 5 compares the voltammetry curves and power density dependence for MEAs with Nafion^®^ 212 and Nafion-NMP. The characteristics of the studied MEAs practically coincide up to a current density of 0.5–0.6 A∙cm^−2^ (Figure 5, Table 3). At current densities i > 0.7 A∙cm^−2^, the MEA with the Nafion-NMP membrane exhibits benefits over Nafion^®^ 212. The increased power of the MEA with Nafion-NMP compared to Nafion^®^ 212 can be explained by the higher ionic conductivity of Nafion-NMP. This results in a 1.5-fold decrease in the internal MEA resistance compared to Nafion^®^ 212 (Table 3). As a result, the maximum power density of the MEA with the Nafion-NMP membrane was 1000 mW∙cm^−2^, which is 27% higher than with Nafion^®^ 212. The performance of the MEA is determined by the contribution of different components: the electrodes, binder, and membrane. Since all these parameters in the studied MEAs were the same, their performance is determined only by the properties of the membrane. The key properties of the membranes for fuel cell applications are their proton conductivity and gas permeability. The conductivity is better for Nafion-NMP membranes, although the hydrogen permeability varies within the error. Thus, the optimization of the properties of perfluorosulfonic acid membranes by changing the method of their preparation leads to an increase in the power of the MEA based on them. The obtained characteristics of the MEA are comparable or higher in relation to the literature data. For example, the P^max^ values for MEAs based on Nafion^®^ 212 and Nafion^®^ 117 membranes were 200 mW·cm^−2^ [49] and 500 mW∙cm^−2^ [50], correspondingly.

## 4. Conclusions

The properties of the membrane recast from Nafion polymer dispersion in *N*-Methyl-2-Pyrrolidone (Nafion-NMP) were compared with Nafion^®^ 212. The Nafion-NMP membrane exhibits improved properties when compared with the Nafion^®^ 212 membrane due to the better interconnectivity of the pores and channels system. The key advantage of the Nafion-NMP membrane is the 30% higher ionic conductivity with a comparable water uptake and a hydrogen permeability. Thus, a Nafion-NMP membrane retains more water at a low RH. The maximum power density of the MEA based on the Nafion-NMP membrane is 27% higher when compared with ones based on Nafion^®^ 212. Thus, the conditions of the formation of Nafion membranes have a great influence on the properties of the membranes and MEAs based on them. A change in the composition of the casting solution of perfluorosulfonic acid polymers allows for obtaining materials with an improved microstructure and transport properties. This approach could be used to raise the performance of the MEAs based on the Nafion-type membrane electrolytes.

## Figures and Tables

**Table 1:** Mechanical properties of membranes kept at RH = 32%.

	Nafion^®^ 212	Nafion-NMP	Nafion–Water–Alcohol Solution *
Young’s modulus, MPa	322 ± 10	268 ± 10	269 ± 9
Proportional limit stress, MPa	11.9 ± 0.5	11.6 ± 0.4	9.6 ± 0.2
Break stress, MPa	24.5 ± 1.1	19.8 ± 0.6	9.6 ± 0.2
Break strain, %	255 ± 6	241 ± 11	41 ± 1

* Membrane obtained via casting procedure from water–alcohol solution of Nafion. Data from [38].

**Table 2:** Data on water uptake, proton conductivity, and hydrogen permeability of the studied membranes.

	Nafion^®^ 212	Nafion-NMP
Water uptake in contact with water, wt.%	25.1 ± 0.2	24.9 ± 0.3
Water uptake at RH = 32%, wt.%	3.6 ± 0.1	4.8 ± 0.1
Proton conductivity in contact with water at t = 30 °C, Ohm^−1^∙cm^−1^	8.7·10^−3^	11.5·10^−3^
H_2_ permeability at RH = 95%, t = 25 °C, cm^2^∙s^−1^	(1.34 ± 0.06)·10^−7^	(1.27 ± 0.04)·10^−7^

**Table 3:** Performance of MEAs at RH = 100%, t = 65 °C: R is a high-frequency resistance, P^max^ is a maximum power density, U (0.5 A∙cm^−2^) is a voltage at current density 0.5 A∙cm^−2^.

Electrolyte	R, mOhm∙cm^2^	P^max^, mW∙cm^−2^	U (0.5 A∙cm^−2^), mV
Nafion^®^ 212	72	790	727
Nafion-NMP	48	1000	727

## Data Availability

Data might be available upon request.
